# *OsProT1* and *OsProT3* Function to Mediate Proline- and γ-aminobutyric acid-specific Transport in Yeast and are Differentially Expressed in Rice (*Oryza sativa* L.)

**DOI:** 10.1186/s12284-019-0341-7

**Published:** 2019-11-09

**Authors:** Jin-Hong Lin, Zhi-Jun Xu, Jia-Shi Peng, Jing Zhao, Guo-Bin Zhang, Jun Xie, Zhen-Xie Yi, Jian-Hua Zhang, Ji-Ming Gong, Neng-Hui Ye, Shuan Meng

**Affiliations:** 1 Southern Regional Collaborative Innovation Center for Grain and Oil Crops in China, College of Agronomy, Hunan Agricultural University, Changsha, 410128 China; 2 Key Laboratory of Ecological Remediation and Safe Utilization of Heavy Metal-Polluted Soils, College of Life Science, Hunan University of Science and Technology, Xiangtan, 411201 China; 3 State Key Laboratory of Crop Biology, College of Agronomy, Shandong Agricultural University, Taian, 271018 Shandong China; 4 Department of Biology, Hong Kong Baptist University, Kowloon, 999077 Hong Kong; 5 School of Life Sciences and State Key Laboratory of Agrobiotechnology, The Chinese University of Hong Kong, Shatin, 999077 Hong Kong; 6 National Key Laboratory of Plant Molecular Genetics and CAS center for excellence in Molecular Plant Sciences, Shanghai Institute of Plant Physiology and Ecology, Chinese Academy of Sciences, Shanghai, 200032 China

**Keywords:** OsProT1, OsProT3, Proline, GABA, Transporter, Stress tolerance

## Abstract

**Background:**

Proline (Pro) and γ-aminobutyric acid (GABA) play important roles in plant development and stress tolerance. However, the molecular components responsible for the transport of these molecules in rice remain largely unknown.

**Results:**

Here we identified OsProT1 and OsProT3 as functional transporters for Pro and GABA. Transient expression of eGFP-OsProTs in plant protoplasts revealed that both OsProT1 and OsProT3 are localized to the plasma membrane. Ectopic expression in a yeast mutant demonstrated that both OsProT1 and OsProT3 specifically mediate transport of Pro and GABA with affinity for Pro in the low affinity range. qRT-PCR analyses suggested that *OsProT1* was preferentially expressed in leaf sheathes during vegetative growth, while *OsProT3* exhibited relatively high expression levels in several tissues, including nodes, panicles and roots. Interestingly, both *OsProT1* and *OsProT3* were induced by cadmium stress in rice shoots.

**Conclusions:**

Our results suggested that plasma membrane-localized OsProT1 and OsProT3 efficiently transport Pro and GABA when ectopically expressed in yeast and appear to be involved in various physiological processes, including adaption to cadmium stress in rice plants.

## Background

Pro and GABA are key players in plant growth and resistance to stresses. Accumulation of Pro in pollen is crucial for pollen fertility and deficiency of Pro leads to morphological defects in leaves and inflorescences in *Arabidopsis* (Funck et al. 2012; Mattioli et al. 2012; Biancucci et al. 2015; Mattioli et al. 2018). In maize plants, Pro was also observed to contribute to final grain production (Spoljarevic et al. 2011). Moreover, Pro is proven to be a member of major compatible solutes which are highly soluble compounds with low molecular weight and are usually nontoxic even at high concentrations (Ashraf and Foolad 2007). It could provide osmotic effects for plants to cope with stresses including drought, salt, nutrient deficiency, heat, heavy metal toxicity and UV-B radiation (Szabados and Savoure 2010; Per et al. 2017). During stress, Pro content is dramatically elevated by enhanced synthesis and decreased degradation (Szabados and Savoure 2010; Kaur and Asthir 2015). Increased Pro synthesis is mainly attributed to upregulation of P5CS which is a key enzyme for Pro biosynthesis; thus enhancing *P5CS* expression by transgenic approach could effectively improve the ability of plant to tolerate stresses (Kishor et al. 1995; Igarashi et al. 2000).

GABA, a nonproteinogenic amino acid, rapidly accumulates in response to both abiotic and biotic stresses (Shelp et al. 2012; Bown and Shelp 2016). It has been shown that GABA functions in defense against insect herbivory and drought tolerance in *Arabidopsis* (Scholz et al. 2015; Bown and Shelp 2016; Mekonnen et al. 2016). Moreover, GABA can bind Aluminum-Activated Malate Transporter (ALMT) membrane channels and stimulate anion efflux or inhibit anion influx at the tonoplast or plasma membrane during drought-induced stomatal closure (Ramesh et al. 2015; Bown and Shelp 2016). In addition, GABA is considered as a pivotal amino acid in post-pollination fertilization (Biancucci et al. 2015).

The transport processes of Pro were considered to be important for altering Pro content inside plants, as no relation was observed between the accumulation and synthesis of Pro during grapevine berry maturation (Stines et al. 1999). Biosynthesis of Pro occurs mainly in the cytosol and chloroplasts (Szabados and Savoure 2010), but Pro can be detected in the xylem and phloem sap in several plant species (Weibull et al. 1990; Bialczyk et al. 2004; Lehmann et al. 2010), further confirming the cross-membrane and cross-tissue transport of Pro. Moreover, the long-distance transport of Pro in phloem is increased in response to water stress in alfalfa (Girousse et al. 1996), indicating that Pro transport is essential for plants under both normal and stress conditions. Similar observations were also obtained for GABA, as it can be released from mesophyll cells (Chung et al. 1992) and transported through vascular tissues (Shelp et al. 1999).

The transporters responsible for Pro transport have been identified in some subfamilies of the amino acid transporter (AAT) family in *Arabidopsis* including the amino acid permease (AAP) family, the lysine-histidine transporter family and the Pro transporter (ProT) family. Meanwhile, some members of the ProT family and GABA transporters in *Arabidopsis* are also responsible for transporting GABA (Lehmann et al. 2010; Shelp et al. 2012). It is worth mentioning that ProTs frequently display specific transport activity for Pro, glycine betaine and GABA in different species including *Arabidopsis*, tomato, barley and the common bean (Schwacke et al. 1999; Grallath et al. 2005; Fujiwara et al. 2010; Lehmann et al. 2011; Chen et al. 2016).

Previous studies have identified 79 and 85 *AAT* genes in rice genomes (Lu et al. 2012; Zhao et al. 2012). However, the transport activities of the encoded proteins have rarely been examined. As such, the components responsible for Pro and GABA transport in rice remain unclear. As OsProTs are likely to participate in these transport processes and OsProT2 has been characterized as a functional Pro transporter through its expression in *Xenopus* oocytes (Igarashi et al. 2000), we identified and functionally characterized the other two *OsProT* members in order to uncover the details for Pro and GABA transport in rice. Our results demonstrated that plasma membrane-localized OsProT1 and OsProT3 are functional uptake transporters for Pro and GABA in yeast and might get involved in normal growth and stress tolerance *in planta*.

## Results

### Phylogenetic Analysis and Prediction of Transmembrane Helices for OsProT1 and OsProT3

**Fig. 1:**
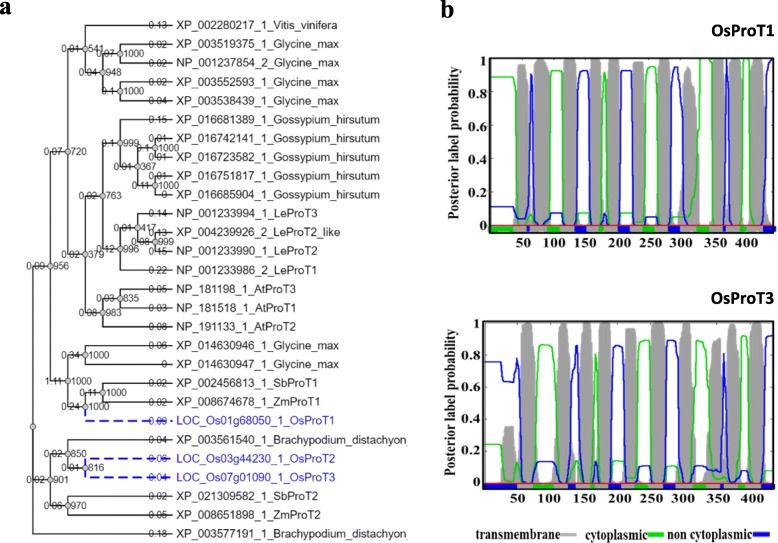
Phylogenetic analysis and prediction of transmembrane helices for OsProT1 and OsProT3**. a** Phylogenetic analysis of ProT protein sequences from *Oryza sativa (Os), Zea mays (Zm), Sorghum bicolor (Sb), Arabidopsis thaliana (At), Lycopersicon esculentum (Le), Brachypodium distachyon, Vitis vinifera, Glycine max and Gossypium hirsutum.* Dendrogram was generated by NGPhylogeny.fr (https://ngphylogeny.fr.) with 1000 bootstrap replications using the default parameters. Values at each tree root indicate bootstrap values and values on each branch indicate branch lengths. **b** Transmembrane prediction of OsProT1 and ProT3 using Phobius (http://phobius.sbc.su.se/)

The ProT subfamily contains three members in rice (Zhao et al. 2012). The OsProT1 sequence exhibits 38.7% and 40% identity to OsProT2 and OsProT3 (Additional file 1: Figure S1), respectively. Surprisingly, a relatively higher identity of 81.4% was found between OsProT2 and OsProT3 (Additional file 1: Figure S1), indicating that OsProT2 and OsProT3 might have similar molecular functions. Phylogenetic analysis of ProTs in *Oryza sativa*, *Zea mays*, *Sorghum bicolor*, *Arabidopsis thaliana*, *Lycopersicon esculentum, Brachypodium distachyon*, *Vitis vinifera*, *Glycine max* and *Gossypium hirsutum* was performed to examine the evolutionary relationships of OsProTs with other ProTs. OsProT1 are closely related with ZmProT1 and SbProT1, while OsProT2 and OsProT3 show closer evolutionary relationship to XP 003561540.1 from *Brachypodium distachyon* than ProTs from other species (Fig. 1a). Additionally, ProT members from *Arabidopsis thaliana*, *Lycopersicon esculentum* and *Gossypium hirsutum* which all belong to dicotyledons can be grouped into three small clusters, and each of these three clusters is specific to one species (Fig. 1a). Noting that only one ProT member existed in *Vitis vinifera* which has not undergone recent whole genome duplication (Jaillon et al. 2007).

Membrane-spanning regions analyses using Phobius (http://phobius.sbc.su.se/) revealed that both OsProT1 and OsProT3 were predicted to contain 11 putative transmembrane regions (Fig. 1b). The regions between 9th and 10th transmembrane segments of OsProT1 and OsProT3 are slightly short.

### OsProT1 and OsProT3 are Localized in the Plasma Membrane

**Fig. 2:**
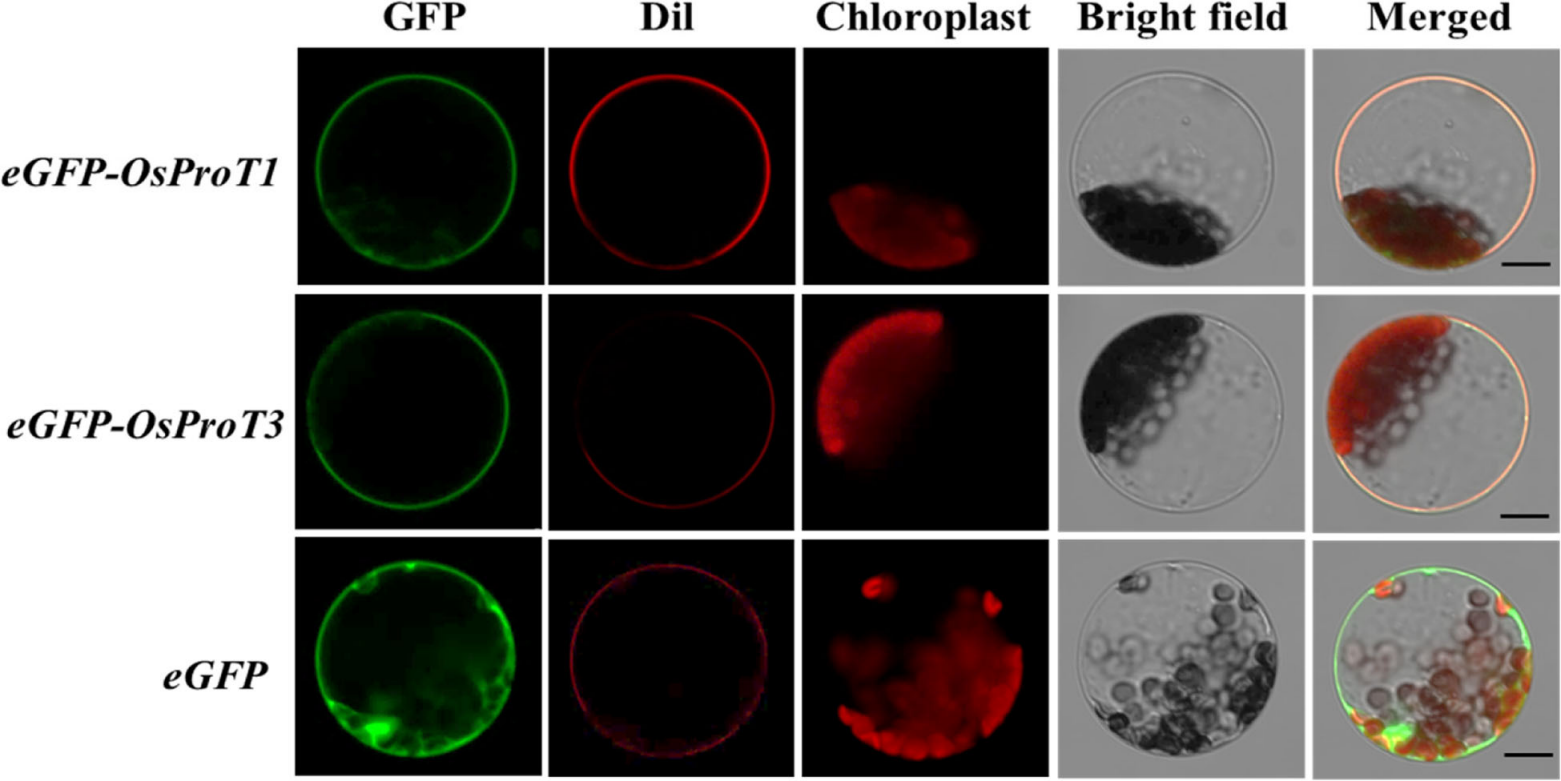
Subcellular localization of eGFP-OsProT fusion proteins in *Arabidopsis* mesophyll protoplasts. *eGFP-OsProT1* or *eGFP-OsProT3* was driven by the 35S promoter and transiently expressed in *Arabidopsis* protoplasts. The fluorescent dye Dil at a concentration of 10 μM was used to indicate the plasma membrane. Bars = 10 μm

Prediction of membrane-spanning regions of OsProT1 and OsProT3 indicated that these two proteins are both localized to membranes. To confirm whether OsProT1 and OsProT3 are targeted to the plasma membrane or to subcellular membranes, eGFP fusion proteins driven by the 35S promoter were transiently expressed in mesophyll protoplasts of *Arabidopsis*. As shown in Fig. 2, GFP fluorescence of eGFP-OsProT1 or eGFP-OsProT3 was observed at the plasma membrane and co-localizes with the plasma membrane marker Dil (1,1′-dioctadecyl-3,3,3′,3′-tetramethylindocarbocyanine perchlorate). These data demonstrate that OsProT1 and OsProT3 are localized to the plasma membrane.

### OsProT1 and OsProT3 are Functional Transporters for pro and GABA in Yeast

**Fig. 3:**
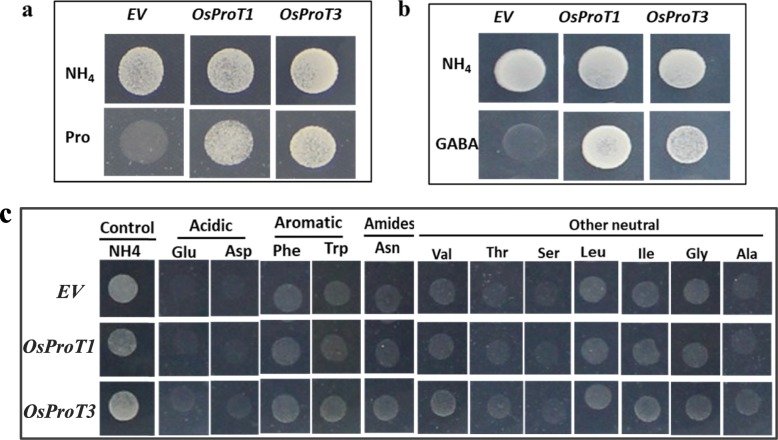
Functional analyses of OsProT1 and OsProT3 in yeast mutant *22△10α*. **a-c,** Yeast mutant strain *22△10α* was transformed with *p426GPD* empty vector (*EV*) or the plasmid containing the coding sequence of *OsProT1* or *OsProT3*, and grown on SD plates with (NH_4_)_2_SO_4_, Pro, GABA or other amino acid as the sole nitrogen source for about 4 days

To investigate the transport activities of OsProT1 and OsProT3, functional complementation experiments were performed using the yeast amino acid transport mutant *22△10α*. The *22△10α* yeast strain lacks ten amino acid transporters addressed at the plasma membrane and is unable to grow on any proteinogenic amino acid or GABA as the sole nitrogen source, except for arginine (Besnard et al. 2016). OsProT1 and OsProT3 were expressed in *22△10α* yeast under the control of the GPD promoter; transformants expressing OsProT1 or OsProT3 were able to grow on media with Pro or GABA as the sole nitrogen resource (Fig. 3a and b), suggesting of that OsProT1 and OsProT3 have the ability to uptake Pro or GABA into yeast cells. Interestingly, *22△10α* expressing OsProT1 or OsProT3 failed to restore the growth defect on media with other amino acids as the sole nitrogen resource, including acidic (Asp or Glu), aromatic (Phe, Trp, or Asn), or other neutral (Val, Thr, Ser, Leu, Ile, Gly, or Ala) amino acids (Fig. 3c). These results suggested that OsProT1 and OsProT3 are functional transporters and specifically transport Pro and GABA.

**Fig. 4:**
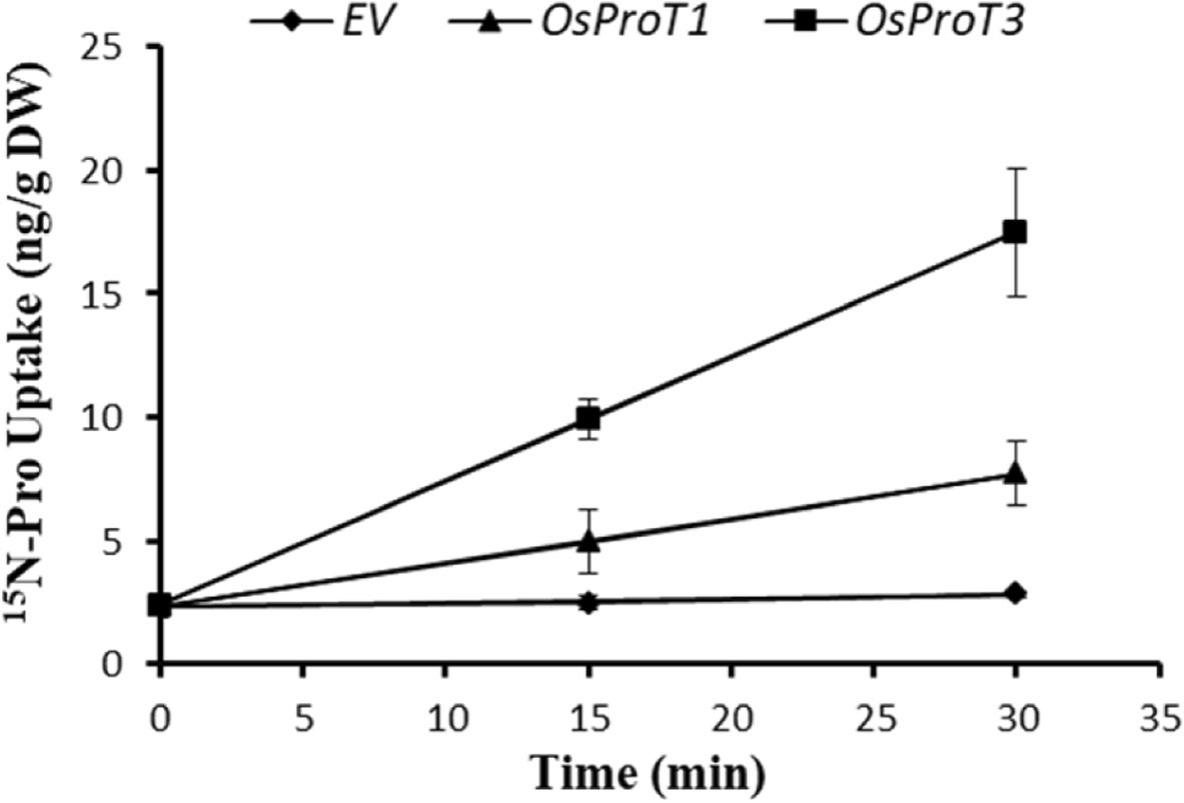
Uptake of ^15^N-labeled Pro by yeast *22△10α* cells. Yeast cells expressing *OsProT1* or *OsProT3* were incubated with 1.5 mM ^15^N-Pro for 15 min and 30 min, and the ^15^N retained in the cells was subsequently measured. Values are means ± SD, *n* = 4

The functions of OsProT1 and OsProT3 in the uptake of Pro were further confirmed by ^15^N-Pro labeled experiments. Yeast *22△10α* cells expressing OsProT1, OsProT3 or the empty vector (*EV*) were cultured with ^15^N labeled Pro, and the ^15^N content in yeast cells was subsequently analyzed. As shown in Fig. 4, *22△10α* cells expressing OsProT3 possessed significantly higher ^15^N content than the cells expressing OsProT1, and both were higher than that in cells transformed with *EV* 15 min or 30 min after labeling (Fig. 4), indicating that both OsProT1 and OsProT3 are Pro transporters for absorbing Pro into cells and that OsProT3 is more capable than OsProT1 in transporting Pro when expressed in yeast.

**Fig. 5:**
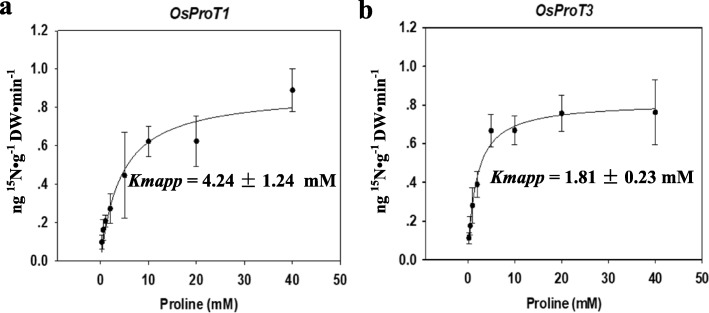
OsProT1 and OsProT3 are low affinity Pro transporters. **a** and **b,** *OsProT1* (a) or *OsProT3* (b) expressed yeast strain *22△10α* was cultivated with different concentrations of ^15^N-labeled Pro for 10 min and the ^15^N content was subsequently determined after being collected and dried. *Kmapp* values were calculated by fitting to the Michaelis-Menten equation in the SigmaPlot 12.0 program. Data are means ± SD, *n* = 4

To determine the affinity of OsProT1 and OsProT3 for Pro, concentration-dependent ^15^N labeled Pro uptake by *22△10α* expressing *OsProT1* and *OsProT3* were performed. Apparent *Km (Kmapp)* values were determined by fitting ^15^N uptake rates at each substrate concentration to the Michaelis-Menten equation. As shown in Fig. 5, *Kmapp* values of OsProT1 and OsProT3 for Pro are 4.24 ± 1.24 mM and 1.81 ± 0.23 mM, respectively. These data indicated that OsProT1 and OsProT3 are both in the low-affinity range for transport of Pro, while OsProT3 showed a higher affinity than OsProT1 with respect to Pro transport.

### *OsProT1* and *OsProT3* showed Differential Expression Patterns in Rice

**Fig. 6:**
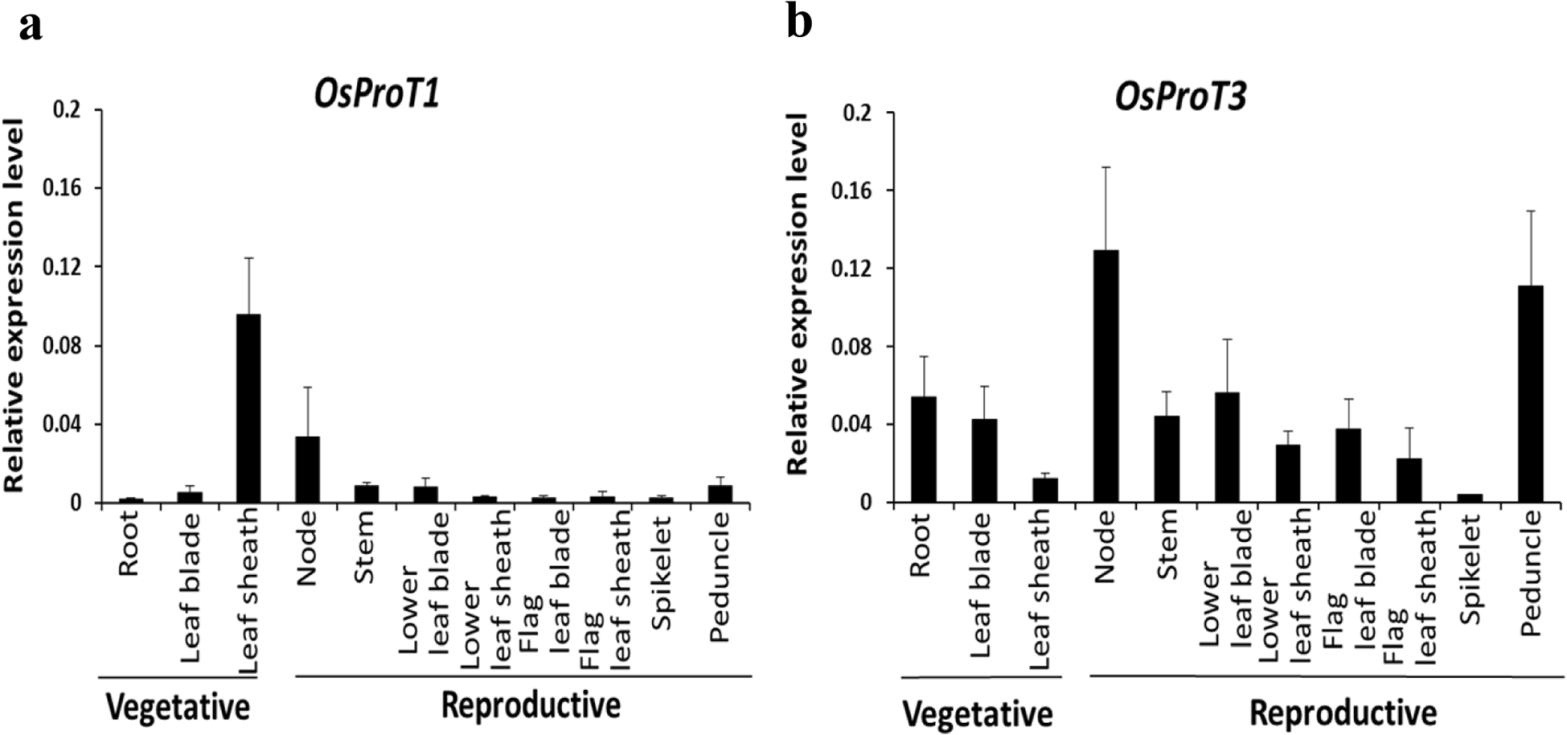
Expression levels of *OsProT1* and *OsProT3* in different organs at vegetative and reproductive stages. **a** and **b**, Different samples were collected from rice grown in the soil. The expression levels of *OsProT1* (**a**) and *OsProT3* (**b**) were determined by real-time RT-PCR using the *OsACTIN1* gene as internal control. Values are means ± SD, *n* = 4

To evaluate the physiological function of *OsProT1* and *OsProT3*, their expression levels in different tissues at vegetative and reproductive stages were detected. *OsProT1* exhibited highest expression in the leaf sheath at the vegetative stage with higher expression in nodes at the reproductive stage than in other tissues (Fig. 6a). *OsProT3* showed relatively high expression levels in several tissues including root and leaf blade with higher levels in nodes and peduncles (Fig. 6b). *OsProT2* was predominantly expressed in the leaf blade, especially in the flag leaf blade at the reproductive stage (Additional sfile 1: Figure S2). These data indicated that *OsProT* members are expressed differentially within rice plant and might be involved in different transport processes for Pro and GABA.

**Fig. 7:**
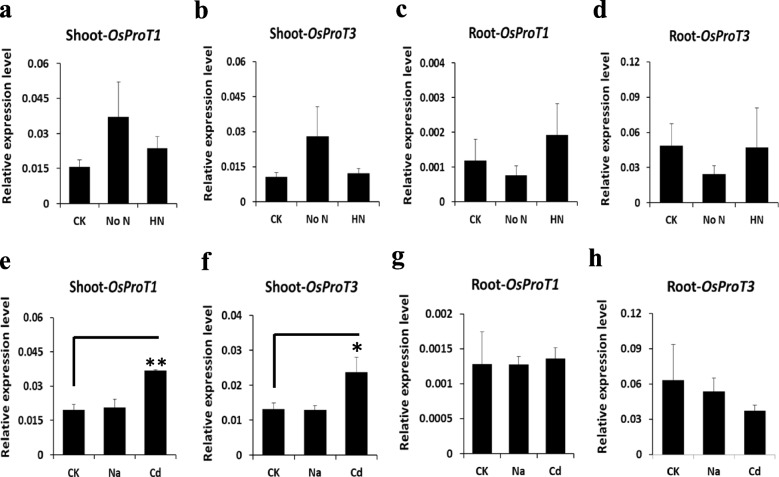
Expression levels of *OsProTs* in response to different abiotic stresses. **a**-**d**, *OsProT1* (**a, c**) and *OsProT3* (**b, d**)  transcript levels in response to nitrogen deficiency or high nitrogen treatment. **e**-**h**, Expression levels of *OsProT1* (**e, g**) and *OsProT3* (**f, h**) in response to salt stress or cadmium treatment. Rice plants were grown hydroponically for about 2 weeks using Yashida solution, and then exposed to Yoshida solution (CK) or Yoshida solution without NH_4_NO_3_ (No N), with 10 mM NH_4_NO_3_ (HN), with 50 μM CdCl_2_ (Cd) or with 100 mM NaCl (Na) for 2 days. Roots and shoots were harvested for RNA extraction and detection of *OsProTs* expression level. Values are means ± SD, *n* = 3. Statistical significance was tested by two-tailed Student’s t tests. Differences were dseemed significant at *P* < 0.05 (*) and extremely significant at *P* < 0.01 (**)

Given that abiotic stresses affect the expression of *ProT* genes in many species, including *Arabidopsis*, common bean and barley (Rentsch et al. 1996; Ueda et al. 2001; Lehmann et al. 2010; Chen et al. 2016), we further characterized the expression levels of *OsProTs* in response to diverse abiotic stresses. Nitrogen deficiency or high nitrogen treatment exerted only a weak influence on *OsProTs* expressions, except for *OsProT2*, which was significantly repressed in roots by nitrogen deficiency (Fig. 7a-d, Additional file 1: Figure S3a and b). The expressions of *OsProT1* and *OsProT3* in shoots, but not in roots, were significantly enhanced by cadmium treatment (Fig. 7e and f). However, cadmium stress exerted very weak effects on *OsProT2* expression (Additional file 1: Figure S3c and d), and salt stress hardly affects the expression of all *OsProTs* in both the shoots and roots (Fig. 7e-h, Additional file 1: Figure S3c and d). These data suggested that transcript regulation of *OsProTs* might be involved in coping with nutrient and heavy metal stress in rice plants.

## Discussion

The accumulation of plant compatible osmolytes, such as Pro and GABA, in response to stress is frequently observed, which is mainly attributed to a combination of increased synthesis and decreased degradation (Lehmann et al. 2010; Shelp et al. 2012; Kaur and Asthir 2015). Meanwhile, abundant evidence has proven that transport of these compatible osmolytes also plays important roles (Igarashi et al. 2000; Lehmann et al. 2010; Shelp et al. 2012). However, the transporters involved in transporting Pro and GABA are largely unknown.

A previous study reported that OsProT2 transports Pro when expressed in *Xenopus* oocytes (Igarashi et al. 2000). In this study, we characterized the other two OsProT members, OsProT1 and OsProT3, as functional transporters for Pro and GABA. AtProT1–3, HvProT2 and LeProTl were previously identified as efficient transporters for Pro, glycine betaine and GABA (Schwacke et al. 1999; Grallath et al. 2005; Fujiwara et al. 2010). Rice lacks the enzyme requirement for glycine betaine biosynthesis; thus, rice likely does not accumulate and transport glycine betaine (Rathinasabapathi et al. 1993). When expressed in yeast, OsProT1 and OsProT3 specifically mediated the transport of Pro and GABA (Figs. 3, 4 and 5). However, the affinity for Pro was different between the individual transporters (Fig. 5). OsProT3 showed a higher affinity for Pro than that of OsProT1, and they are both lower than that of the three AtProTs with *Kmapp* values of 0.427 ± 0.017 mM, 0.500 ± 0.005 mM, 0.999 ± 0.036 mM for Pro, respectively (Grallath et al. 2005).

OsProT1 and OsProT3 are both localized to the plasma membrane and function to uptake Pro and GABA from the apoplast (Fig. 2, 3 and 4). Further expression analyses indicated that OsProT1, OsProT3 and OsProT2 play different roles within rice plants. The high expression of *OsProT1* in leaf sheath at the vegetative stage (Fig. 6a) suggests that OsProT1 might uptake Pro and GABA as nutrition for seeding growth. OsProT2 might be involved in Pro and GABA remobilization from flag leaf blade, as it shows much higher expression in flag leaf blade than in other tissues (Additional file 1: Figure S2). OsProT3 likely contributes to more physiological processes due to its high expression in more tissues at different stages (Fig. 6b). Consistently, ProTs in *Arabidopsis* also exhibit similar substrate specificity but different expression patterns (Grallath et al. 2005). *AtProT1* is expressed mainly in phloem or phloem parenchyma throughout the whole plant, while *AtProT2* is localized in the roots, and *AtProT3* is expressed in the above-ground portions of plants (Grallath et al. 2005; Lehmann et al. 2011). In addition to *ProTs*, evidence indicates that *AtAAP* members also exhibit difference in tissue expression patterns which guarantees their unique functions in *Arabidopsis* (Tegeder and Rentsch 2010; Tegeder 2012; Tegeder and Masclaux-Daubresse 2018).

Upon exposure to salt, plants often accumulate Pro (Ueda et al. 2001; Mansour and Ali 2017; Per et al. 2017). However, gene expression data revealed that salinity hardly affects expression levels of *OsProTs* (Fig. 7e-h, Additional file 1: Figure S3c and d). Consistent with our results, *OsProT2* was not induced by salt treatment in a previous study (Igarashi et al. 2000). Surprisingly, Cd stress induced the expression of *OsProT1* and *OsProT3* in shoots (Fig. 7e and f). The accumulation of Pro frequently occurs in response to heavy metal stress (Sharma and Dietz 2006; Amna et al. 2015), and high constitutive Pro content was detected in metal-tolerant plants (Sharma and Dietz 2006). Given that the transport of Pro might participate in Cd stress tolerance, as the expression of *OsProT1* and *OsProT3* in shoots were induced by Cd, additional investigation into Pro transport process may improve plant tolerance to Cd.

## Conclusion

In the present study, two plasma membrane-localized OsProT members, OsProT1 and OsProT3, were characterized as functional molecular components for transporting Pro and GABA. When ectopically expressed in yeast, OsProT1 and OsProT3 specifically mediated the uptake of Pro and GABA, and both exhibited low affinity for transporting Pro. *OsProT1* and *OsProT3* might function in differentially physiological processes, including stress tolerance, based on their differential expression patterns. Our work identified new molecular components for controlling the transport of Pro and GABA, and provides new clues to improve stress tolerance in rice by manipulating these transporters.

## Materials and methods

### Plant material and growth conditions

Uniformly germinated seeds (*Oryza sativa* L. var. Nipponbare) were cultivated in 96-well plates with removed bottoms (Li et al. 2015) and were then grown hydroponically in Yoshida solution (Adjust pH to 5.8, refreshed every 2 d) at 28 °C, approximately 60% humidity and a 16-h-light/8-h-dark photoperiod. 14-day-old seedlings were further treated for the analysis of gene expression. Yoshida solution without NH_4_NO_3_ or containing 10 mM NH_4_NO_3_ was used for nitrogen-starvation or high-nitrogen experiments, respectively. Yoshida solution with 50 μM CdCl_2_ or 100 mM NaCl was used for abiotic stress treatments.

To determine gene expression at the reproductive stage, 3-week-old rice seedlings were transplanted into flooded soil in pots with two seedlings per hill. Soil properties were pH =6.66, organic matter = 48.51 g·kg^− 1^, total *N* = 2.2 g·kg^− 1^, total *P* = 0.8 g·kg^− 1^, total K = 6.32 g·kg^− 1^, available *N* = 123.24 mg·kg^− 1^, available *P* = 26.04 mg·kg^− 1^, and available K = 118.19 mg·kg^− 1^. Fertilizers used were urea for N at dose of 34.5 g·m^− 2^, single superphosphate for P at dose of 50 g·m^− 2^, and potassium chloride for K at doses of 20 g·m^− 2^. Different tissues at reproductive stage were collected for further RNA extraction.

### Radial tree construction and multiple alignments of OsProTs

OsProT protein sequences were obtained (http://rice.plantbiology.msu.edu/) according to accession numbers LOC_Os01g68050.1 for OsProT1, LOC_Os03g44230.1 for OsProT2, and LOC_Os07g01090.1 for OsProT3 and were then aligned using CLUST W with default parameters. The homologous sequences in other species were obtained via blasting on the NCBI database using OsProT sequences as references. The phylogenetic tree of ProT proteins was constructed by NGPhylogeny.fr (Lemoine et al. 2019) with 1000 bootstrap replications using the default parameters after removing redundant sequences and selecting representative splice forms of the same gene.

### Functional Analysis of OsProTs in *22△10α*

Coding sequences of *OsProT1* and *OsProT3* were amplified (primers are listed in Additional file 1: Table S1) from cDNA template of Nipponbare followed by cloning into the p426GPD vector with the *BamH1* and *EcoR1* restriction sites, leading to the final p426GPD-OsProT1 and p426GPD-OsProT3 constructs, respectively. p426GPD-OsProT1, p426GPD-OsProT3 and the empty vector p426GPD were separately transformed into mutant yeast *22△10α* cells as previously described (Elble 1992). Functional complementation assays were performed as described (Fischer et al. 2002; Besnard et al. 2016) with minor modifications. Yeast cells were grown to log phase using SD medium containing 3 mM (NH_4_)_2_SO_4_. Then, cells were collected, rewashed, diluted to an OD600 of 0.8 in sterile water, and spotted onto SD plates containing indicated L-amino acid as the sole nitrogen source at dose of 3 mM. The cells were then grown for about 4 d before being photographed. For ^15^N-Pro uptake assays, 1.5 mM ^15^N labeled L-Pro (99 atom %) was added to the culture after yeast cells had reached log phase, and the cells were collected at 0 min, 15 min and 30 min. Then, the cells were immediately washed three times by ultrapure water and dried at 80 °C for 24 h. ^15^N retained in the cells was measured as previously described (He et al. 2017) using a continuous- flow isotope ratio mass spectrometer coupled to a carbon nitrogen elemental analyzer.

### Determination of *Kmapp* for Pro by Yeast Uptake Assay

Analyses of *Kmapp* were performed as described previously (Hsu et al. 1993; Hirner et al. 2006; Wang and Tsay 2011) with minor modifications. Yeast cells expressing *OsProT1* or *OsProT3* were cultured with SD medium containing 3 mM (NH_4_)_2_SO_4_ until the OD600 reached 0.8. Then, the cells were cultured with SD medium containing different concentrations of ^15^N-Pro ranging from 0.25 mM to 40 mM for 10 min. The collection of cells and determination of ^15^N contents were performed as described above. The curves and the *Kmapp* values were obtained by fitting to the Michaelis-Menten equation in the SigmaPlot 12.0 program.

### Subcellular Localization Analysis of OsProT1 and OsProT3

The amplified fragment of *OsProT1* and *OsProT3* (primers used for amplifying are listed in Additional file 1: Table S1) coding sequences were cloned in frame with eGFP into pA7-eGFP vector using *Xba1* and *BamH1*, resulting in *eGFP-OsProT1* and *eGFP-OsProT3* constructs, respectively, driven by the 35S promoter. Fusion constructions were then transiently expressed in *Arabidopsis* protoplasts as described previously (Yoo et al. 2007). Transformed protoplasts were incubated in the dark at 22 °C overnight, and eGFP images were subsequently obtained using confocal microscopy (Olympus-FV1000) (Peng et al. 2017). The fluorescent dye Dil was used at a concentration of 10 μM to indicate the plasma membrane (Wang et al. 2019).

### Quantitative RT-PCR (Reverse Transcription Polymerase Chain Reaction)

RNA was extracted using TRIzol reagent (Invitrogen, 15,596,026) as previously described (Meng et al. 2016). First-strand cDNA was synthesized using cDNA Synthesis Kit (Vazyme, R212), and quantitative RT–PCR was performed using SYBR Green with a StepOnePlus instrument (Applied Biosystems). *OsACTIN1* (*LOC_Os03g50885.1*) was selected as internal reference gene. Primers used for assays are all listed in Additional file 1: Table S1. The different samples used for the qPCR are biological replicates, and relative expression levels are the expression of *OsProTs* normalized to that of *OsACTIN1* using formula Ratio = 2 ^(*Ct_OsActin − Ct_OsProT*)^.

## Supplementary information

**Additional file 1:** **Figure S1.** Alignment of OsProT proteins. **Figure S2.** Expression levels of *OsProT2* in different organs at vegetative and reproductive stages. **Figure S3.** *OsProT2* transcript levels in rice plants under different abiotic stress conditions. **Table S1.** The primers used in this study.

## Data Availability

All data generated or analyzed during this study are included in this published article and its supplementary information files.

## Abbreviations

AAP: Amino acid permease
AAT: Amino acid transporter
ALMT: Aluminum-Activated Malate Transporter
Dil: 1,1′- dioctadecyl- 3,3,3′,3′- tetramethylindocarbocyanine perchlorate
eGFP: Enhanced green fluorescent protein
EV: Empty vector
GABA: γ-aminobutyric acid
*Kmapp*: Apparent *Km*
Pro: Proline
ProT: Proline transporter
qRT-PCR: Quantitative real time polymerase chain reaction
