# Supplementary material for: OsProT1 and OsProT3 Function to Mediate Proline- and γ-aminobutyric acid-specific Transport in Yeast and are Differentially Expressed in Rice (Oryza sativa L.)
Source: Rice (N Y). 2019 Nov 9;12:79. doi: 10.1186/s12284-019-0341-7 (PMC6842372; doi:10.1186/s12284-019-0341-7)
Supplement: Supplementary file 1 — Additional file 1: Figure S1. Alignment of OsProT proteins. Figure S2. Expression levels of OsProT2 in different organs at vegetative and reproductive stages. Figure S3. OsProT2 transcript levels in rice plants under different abiotic stress conditions. Table S1. The primers used in this study. [file 12284_2019_341_MOESM1_ESM.pptx]

## Slide 1
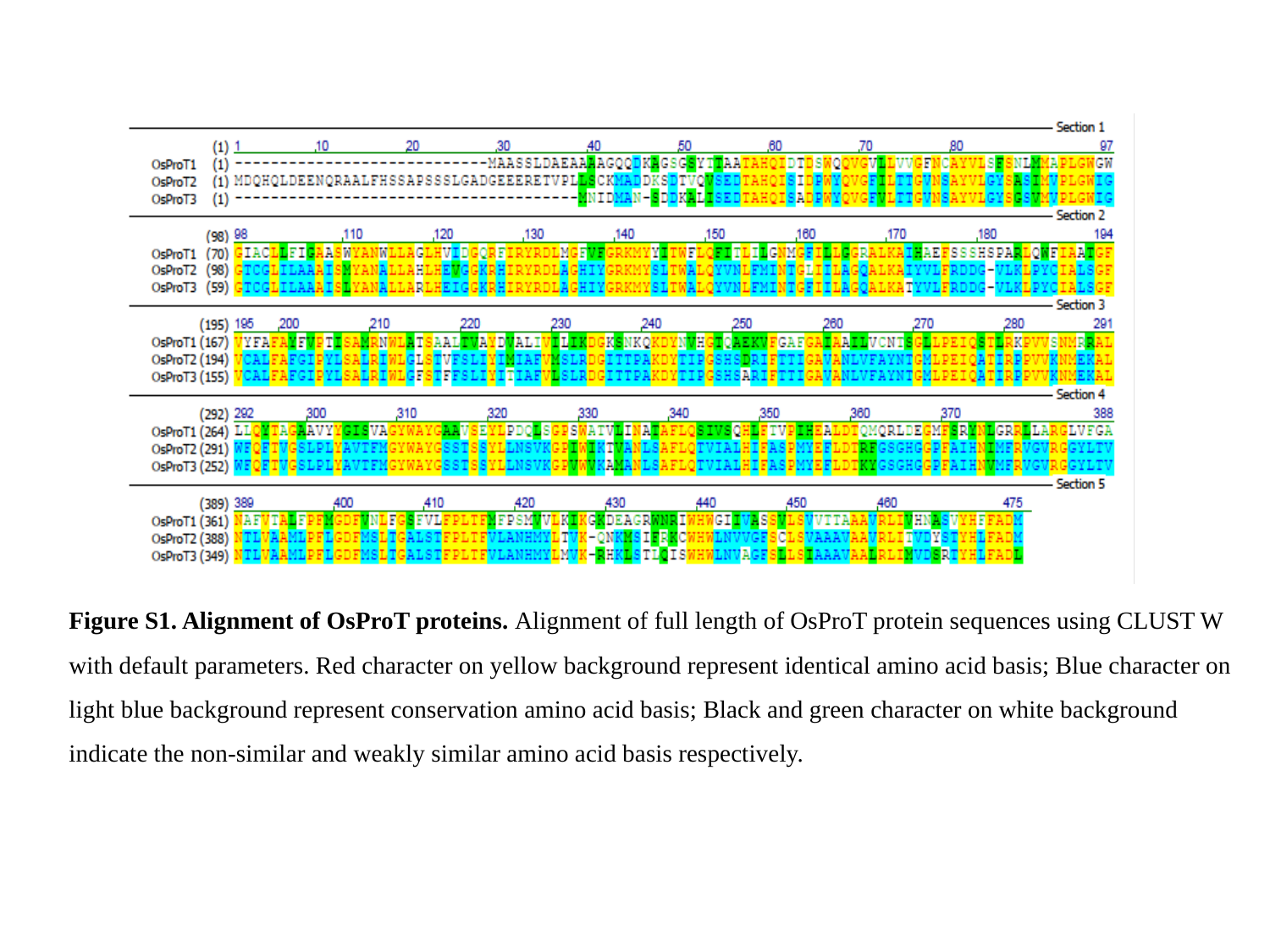

Figure S1. Alignment of OsProT proteins. Alignment of full length of OsProT protein sequences using CLUST W with default parameters. Red character on yellow background represent identical amino acid basis; Blue character on light blue background represent conservation amino acid basis; Black and green character on white background indicate the non-similar and weakly similar amino acid basis respectively.

## Slide 2
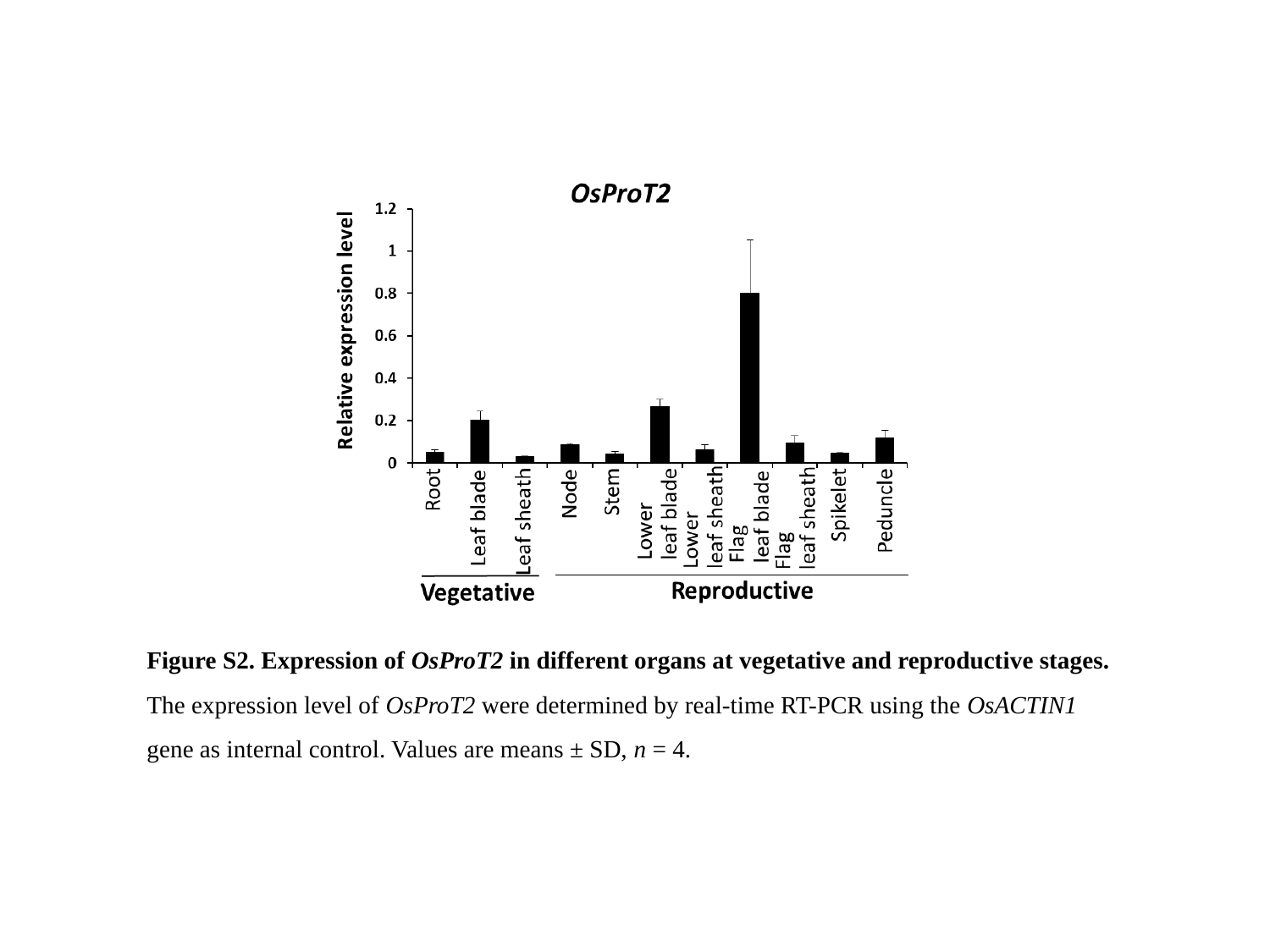

Figure S2. Expression of OsProT2 in different organs at vegetative and reproductive stages.
The expression level of OsProT2 were determined by real-time RT-PCR using the OsACTIN1 gene as internal control. Values are means ± SD, n = 4.

## Slide 3
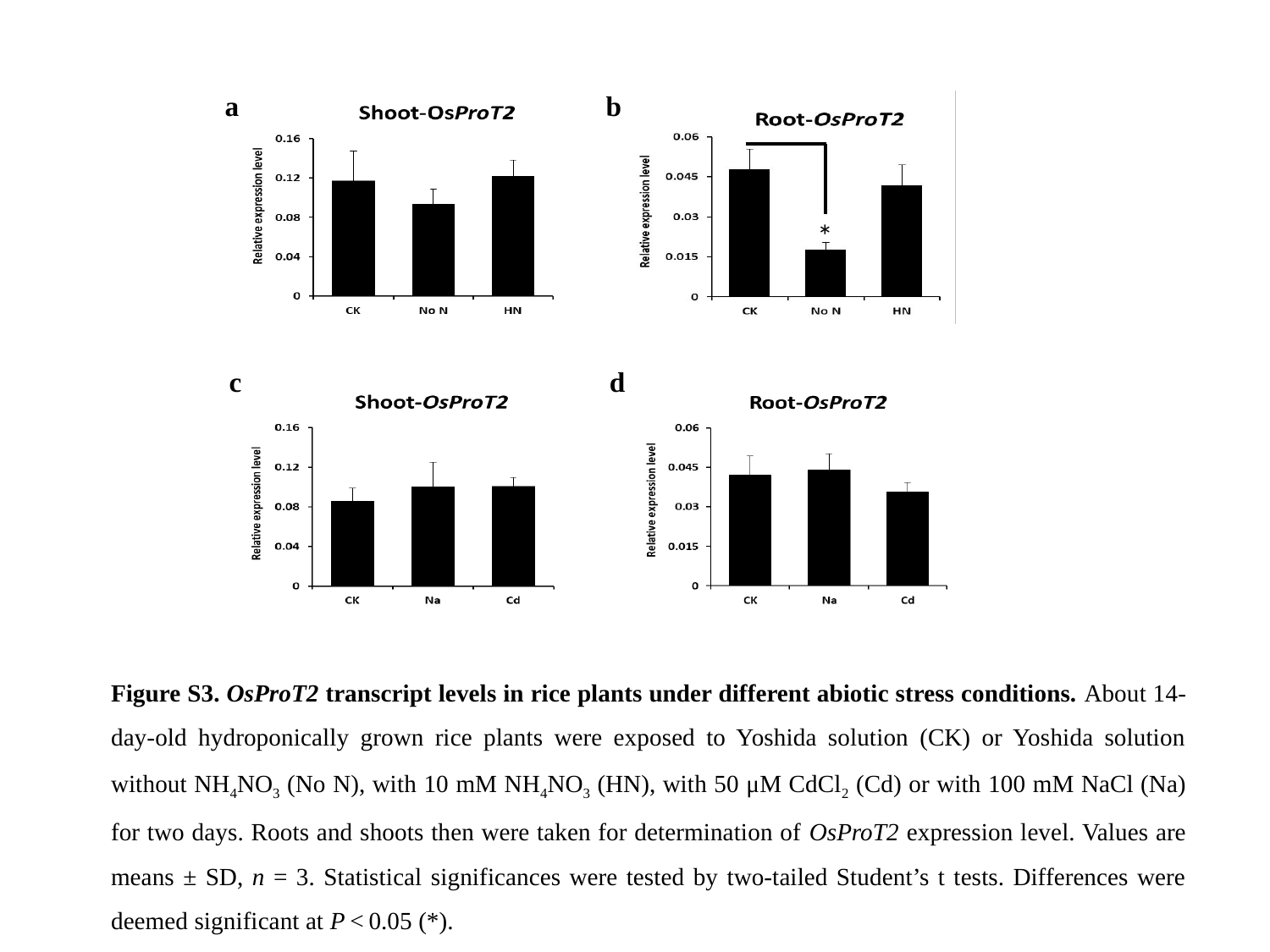

a
 b
 c
 d
*
Figure S3. OsProT2 transcript levels in rice plants under different abiotic stress conditions. About 14-day-old hydroponically grown rice plants were exposed to Yoshida solution (CK) or Yoshida solution without NH4NO3 (No N), with 10 mM NH4NO3 (HN), with 50 μM CdCl2 (Cd) or with 100 mM NaCl (Na) for two days. Roots and shoots then were taken for determination of OsProT2 expression level. Values are means ± SD, n = 3. Statistical significances were tested by two-tailed Student’s t tests. Differences were deemed significant at P < 0.05 (*).

## Slide 4
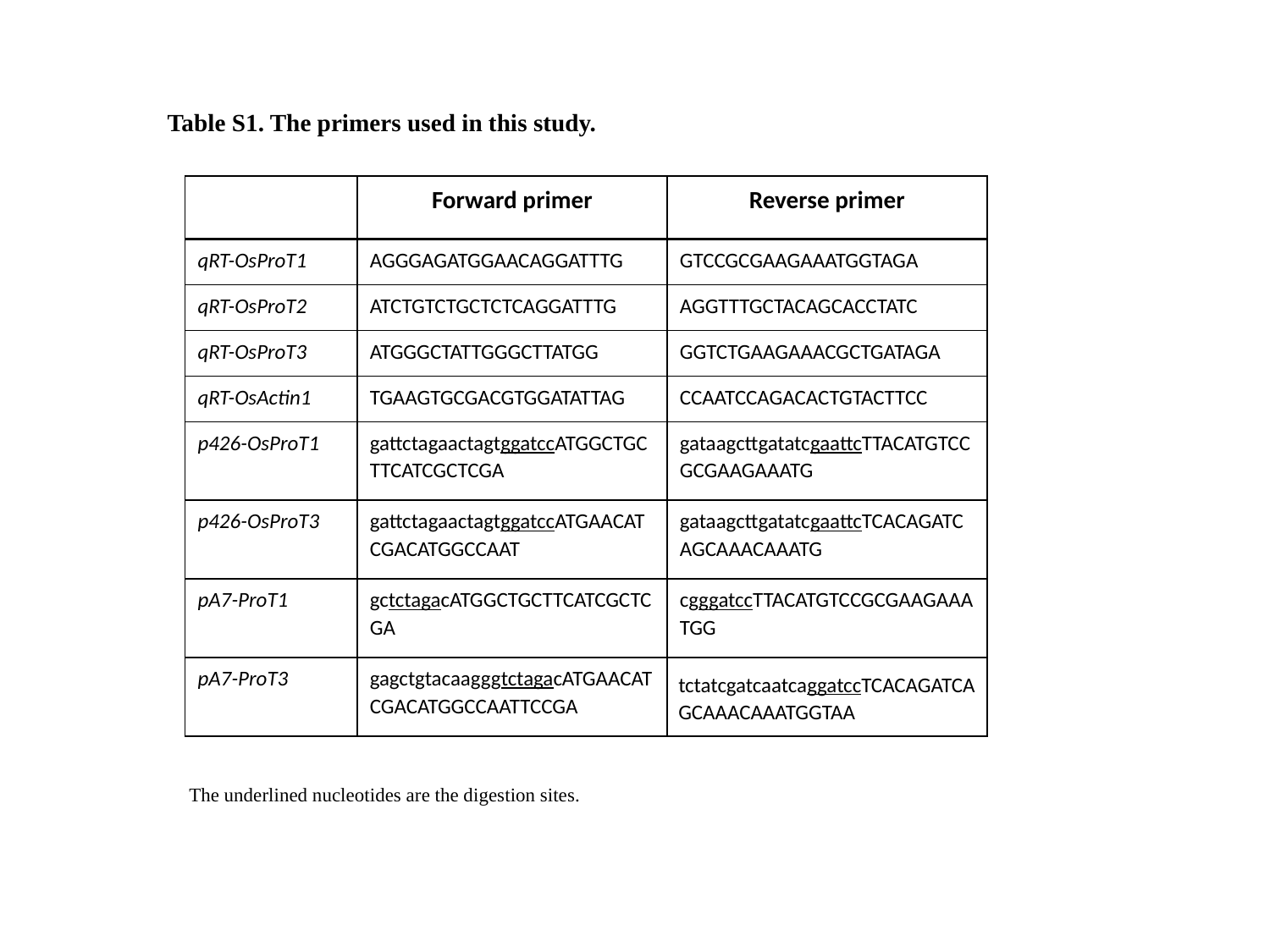

Table S1. The primers used in this study.
| | Forward primer | Reverse primer |
| --- | --- | --- |
| qRT-OsProT1 | AGGGAGATGGAACAGGATTTG | GTCCGCGAAGAAATGGTAGA |
| qRT-OsProT2 | ATCTGTCTGCTCTCAGGATTTG | AGGTTTGCTACAGCACCTATC |
| qRT-OsProT3 | ATGGGCTATTGGGCTTATGG | GGTCTGAAGAAACGCTGATAGA |
| qRT-OsActin1 | TGAAGTGCGACGTGGATATTAG | CCAATCCAGACACTGTACTTCC |
| p426-OsProT1 | gattctagaactagtggatccATGGCTGCTTCATCGCTCGA | gataagcttgatatcgaattcTTACATGTCCGCGAAGAAATG |
| p426-OsProT3 | gattctagaactagtggatccATGAACATCGACATGGCCAAT | gataagcttgatatcgaattcTCACAGATCAGCAAACAAATG |
| pA7-ProT1 | gctctagacATGGCTGCTTCATCGCTCGA | cgggatccTTACATGTCCGCGAAGAAATGG |
| pA7-ProT3 | gagctgtacaagggtctagacATGAACATCGACATGGCCAATTCCGA | tctatcgatcaatcaggatccTCACAGATCAGCAAACAAATGGTAA |
The underlined nucleotides are the digestion sites.
